# Digital health intervention for children with ADHD to improve mental health intervention, patient experiences, and outcomes: a study protocol

**DOI:** 10.1186/s44247-024-00134-4

**Published:** 2024-11-05

**Authors:** Nancy Herrera, Franceli L. Cibrian, Lucas M. Silva, Jesus Armando Beltran, Sabrina E. B. Schuck, Gillian R. Hayes, Kimberley D. Lakes

**Affiliations:** 1 Department of Psychiatry and Neuroscience, School of Medicine, University of California Riverside, 3390 University Ave., Suite 115, Riverside, CA 92501 USA; 2 Fowler School of Engineering, Chapman University, Orange, CA USA; 3 Computer Science Department, University of Iowa, Iowa City, Iowa USA; 4 Department of Computer Science, California State University, Los Angeles, CA USA; 5 Department of Pediatrics, School of Medicine, University of California, Irvine, CA USA; 6 Department of Informatics, Donald Bren School of Information and Computer Science, University of California, Irvine, USA

**Keywords:** ADHD, Protocol, DHI, Digital health intervention, Children, Families, Self-regulation, Intervention, Treatment

## Abstract

**Background:**

Attention Deficit Hyperactivity Disorder (ADHD) is the most prevalent childhood psychiatric condition with profound public health, personal, and family consequences. ADHD requires comprehensive treatment; however, lack of communication and integration across multiple points of care is a substantial barrier to progress. Given the chronic and pervasive challenges associated with ADHD, innovative approaches are crucial. We developed the digital health intervention (DHI)—CoolTaCo [Cool Technology Assisting Co-regulation] to address these critical barriers. CoolTaCo uses Patient-Centered Digital Healthcare Technologies (PC-DHT) to promote co-regulation (child/parent), capture patient data, support efficient healthcare delivery, enhance patient engagement, and facilitate shared decision-making, thereby improving access to timely and targeted mental health intervention for children at significant risk for poor outcomes. The present paper will describe our planned protocol to evaluate the efficacy of CoolTaCo via randomized control trial (RCT).

**Methods/design:**

We will recruit 60 children (ages 8–12) with ADHD who will be randomized to either immediate (*n* = 30) or delayed (*n* = 30) treatment (i.e., a waitlist control group). Among those randomized to immediate treatment, half will be assigned to DHI (delivered via a smartwatch and smartphone application), the other half to an active control treatment as usual (TAU). Unlike the DHI group, the TAU group will receive the smartwatch with no assigned activities, applications, or interventions on the devices. The intervention period will last 16 weeks; after a participant has been in the delayed treatment group for 16 weeks and has completed the post-waiting period assessment, they will be randomly assigned to either the intervention or active control group. Thus, 30 participants will complete the intervention, and 30 will complete the active control, with half of the total sample completing a waitlist period.

**Discussion:**

Individuals with ADHD have complex needs. Despite improvement in outcomes following cognitive behavioral therapies (CBT) and pharmaceutical treatment, long-term maintenance is a challenge often not addressed by traditional medical approaches, and, as we described, ineffective approaches to information sharing across points of care create further barriers to progress. Our research will fill a significant gap in translating early treatment investments and gains into long-term, sustainable outcomes.

This study was registered as a clinic trial at ClinicalTrials.gov (Digital Health Intervention for Children With ADHD, ID# NCT06456372) on 06/13/2024.

## Background

Attention Deficit Hyperactivity Disorder (ADHD) is the most prevalent and widely recognized psychiatric condition affecting children and adolescents, marked by symptoms of inattention, hyperactivity, and impulsivity [1]. Data from the 2016–2019 US National Survey of Children’s Health (NSCH) (2016–2019) revealed that an estimated 6 million (9.8%) US children aged 3–17 years have been diagnosed with ADHD [2]. ADHD has a significant impact on academic performance, social relationships, emotional well-being, behavior, and family dynamics and can co-occur with conditions such as mood disorders and learning disabilities [3, 4]. Therefore, ADHD treatment necessitates long-term maintenance and self-regulation [5]. ADHD requires comprehensive treatment and typically involves multiple points of care such as primary care clinics (e.g., pediatricians), mental health clinics (e.g., psychiatrists, psychologists, and other therapists), and homes and schools (e.g., special education services) [6]. Consistent and effective communication across points of care is essential to help children effectively manage their ADHD. However, such consistent communication is rare [7]. Despite decades of research on evidence-based medical and behavioral treatments, adherence to treatment is a considerable challenge [8].

Furthermore, long-term maintenance is challenging and presents a second significant barrier to progress that is not met by current approaches [9, 10]. Because ADHD challenges are, for most individuals, persistent and lifelong, substantial self-regulation is needed to support initial treatment gains [11, 12]. Moreover, as children with ADHD become adolescents, they often receive less frequent treatment and support for their difficulties despite facing increasingly challenging situations in academic (high school or college curriculum) [13], social settings (exposure to illicit drugs, alcohol), and interpersonal relationships (e.g., family, romantic, friendship) [14]. Research has shown that adolescents with persistent ADHD are more likely to engage in serious risk behaviors (e.g., underage drinking, reckless driving, and illegal drug use) [15, 16]. Self-regulation is a robust predictor of positive outcomes, and late childhood to early adolescence is a critical period for promoting self-regulation to prevent life-altering mistakes rooted in high school years [17].

Given the chronic and pervasive challenges associated with ADHD, innovative approaches for supporting children and adolescents are crucial [18, 19]. Providing assistance and reinforcement with wearable and mobile technologies offers promising results for children and adolescents [20]. Digital Health Interventions (DHIs) can promote self-regulation (which includes self-monitoring) of health behaviors and facilitate more timely and accurate information sharing between multiple points of care. DHIs could substantially alter the current paradigm, leading to long-term improvements in ADHD outcomes [18, 21]. These technologies, however, have traditionally been designed for individual users and without substantial input from children with ADHD and their families [22, 23]. Our goal is to evaluate an evidence-based and integrated (smartwatch and smartphone) DHI that can increase parent–child support and multidisciplinary communication [24]. *The present paper describes our planned protocol to evaluate the efficacy of the DHI CoolTacCo [Cool Technology Assisting Co-regulation] *via* randomized controlled trial (RCT).*

## Methods/design

### Preliminary work influencing current study protocol

With funding from the Agency for Healthcare Research and Quality, we developed and piloted a DHI called CoolCraig, a wearable and connected system combining a smartwatch and a mobile phone application that provides self-and co-regulation strategies to support caregivers and children with ADHD [8, 18, 21, 25–28]. Our research showed that CoolCraig could support self-regulation in children and co-regulation processes between parent and child. The results also revealed a need for improved instructional guidance and coordination across the multiple points of care [29, 30]. Such results informed our goal of expanding the CoolCraig system and developing the DHI, CoolTaCo [Cool Technology Assisting Co-regulation].

### CoolTaCo

The CoolTaCo System will include a smartwatch app for the child and smartphone app for the parent. As a platform to develop the smartwatch and phone application, we will use the Apple digital ecosystem (i.e., Apple Watch and iPhone). In this manner, we leverage existing Apple Watch features to support intervention, collect and report data (e.g., calendar reminders, activity, sleep, and heart rate data collection and reporting), and a parent iPhone application paired with the child’s Apple Watch. The application will allow parents to enter goals for children (e.g., goals established in therapy or in collaboration with a child’s teacher), approve goals the children initiate for themselves, and create rewards for their child.

**Fig. 1:**
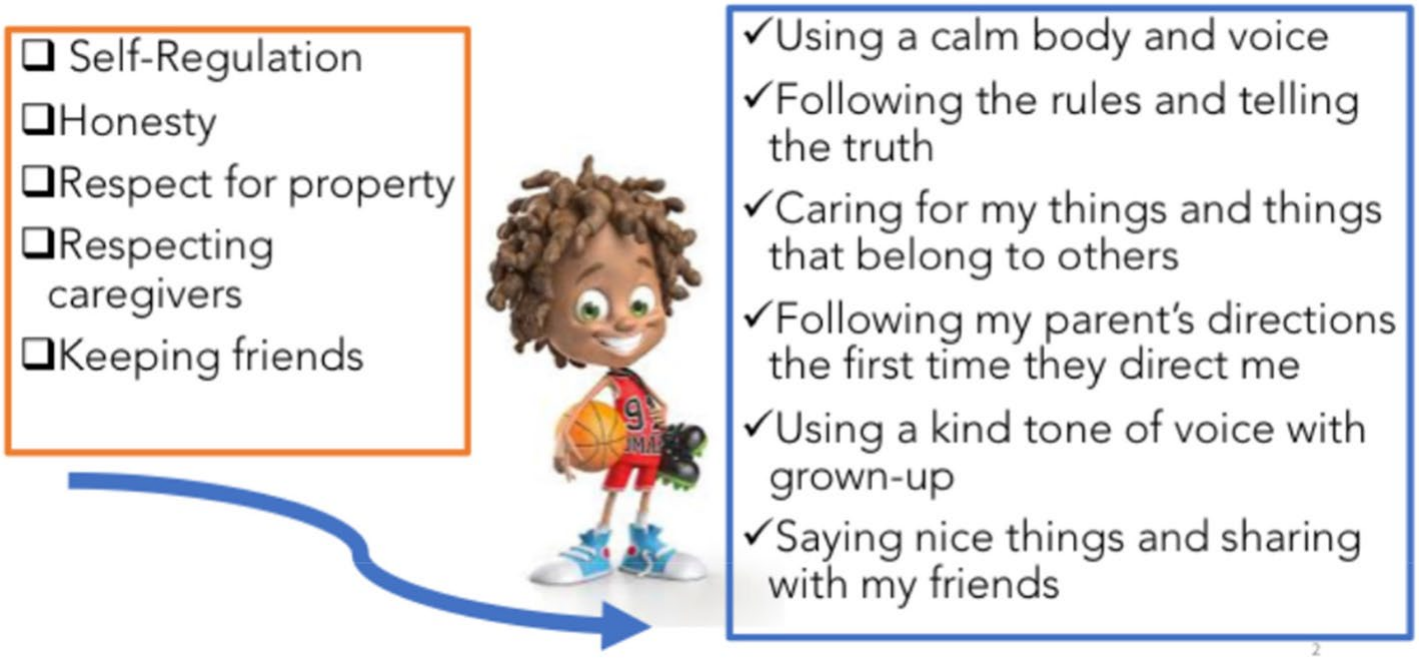
Child mini-lesson example on social skills

Derived from prior ADHD RCTs and implemented in our community programs, CoolTaCo will also include mini-lessons that are created for children and parents on topics such as assertiveness and communication. Key themes of those lessons include ‘Assertion,’ ‘Ignoring Provocation,’ ‘Organization/Executive Function Planning,’ ‘Productivity,’ ‘Self-Regulation,’ and ‘Problem Solving.’ Brief lessons support the key themes with the aim of reducing maladaptive behaviors (aggression, whining/complaining, avoiding non-preferred tasks, disorganization, losing items) and reinforcing the development of an increase in adaptive behaviors necessary for optimizing academic and social outcomes. Figure 1 depicts an example of a mini-lesson which is part of the unit on the social skill of ‘Assertion,’ in which children are prompted to use a self-evaluation strategy when met with a challenge, disappointment, or frustration.

**Fig. 2:**
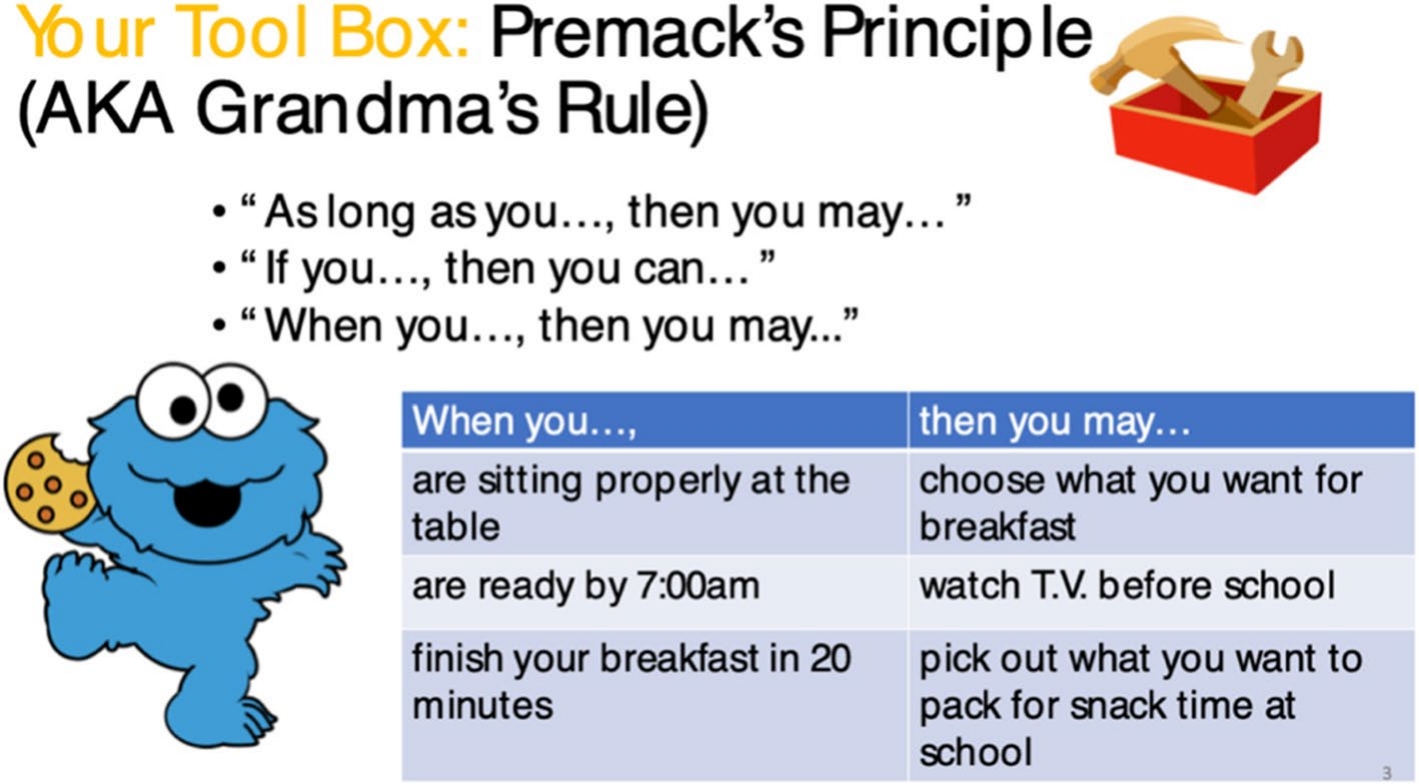
Parent mini-lesson example

The CoolTaco System will also deliver parent interventions derived from evidence-based parent ADHD training programs developed and studied in prior ADHD clinical trials and delivered in our community-based parent education programs. Figure 2 depicts an example of a mini-lesson which is part of the main behavioral parent training unit on using communication strategies in efforts to increase the likelihood that a child will follow directions. In this lesson, parents learn that some communication strategies are more effective in specific settings than others and they learn how to identify when and where to choose from a ‘toolbox’ or variety of different ways of using effective communication as an antecedent for increasing desired behaviors.

The CoolTaco System allow children and caregivers to build self-regulation by setting goals and monitoring and evaluating progress. CoolTaco will also support self-efficacy by helping children and caregivers to reflect on successes and behaviors that need to change. Goals can be set in multiple domains, customized to the treatment plan. In addition to behavioral goals, goals can address hours of sleep per night, amount of physical activity, and medication regimen compliance. Once goals are set, progress can be tracked both daily and weekly. The use of goal-setting, daily self-monitoring, and updating personal goals will promote self-regulation; in addition, it can provide data to be shared with parents and the child’s clinician(s) or educators regarding progress meeting treatment goals. In our clinical practice and research with children with ADHD, common barriers to change include lack of consistency implementing strategies and forgetting to use strategies, both of which are addressed by the system design. CoolTaco will provide an appealing, sustainable intervention that can reinforce positive health behaviors over time.

### RCT design

We will recruit 60 children (ages 8–12) with ADHD who will be randomized to either immediate (*n* = 30) or delayed (*n* = 30) treatment (i.e., a waitlist control group). Among those randomized to immediate treatment, half will be assigned to DHI (an intervention delivered via a smartwatch for the child and smartphone application for the parent), and half will be assigned to an active control [treatment as usual (TAU)] group. Individuals in the TAU group will receive the smartwatch with no assigned activities or applications. The intervention period will last 16 weeks, and participants will complete study assessments before, during, and after the intervention period. Participants randomized to delayed treatment will wait for 16 weeks; they will complete an assessment before and after the waiting period. After this waiting period, they will be randomly assigned to either DHI or TAU. Thus, 30 participants will complete 16 weeks of DHI, and 30 will be followed for 16 weeks of TAU, with half of the total sample also completing a waitlist period.

We will loan smartwatches and phones as necessary for the study in both the DHI and the TAU groups. Participants will be taught basic cybersecurity and privacy best practices, including how to lock and encrypt phone data.

#### Participants

We will recruit children and one of their parents/guardians/caregivers from a local school for children with ADHD and local clinics. If additional parents/guardians/caregivers wish to enroll, their participation will be included as a covariate in our analyses. Participants will also include primary points of care such as educators and clinicians working with the enrolled child participants. 

When a child and parent enroll, we will ask them to indicate the primary points of care for their child. These may include a pediatrician or family doctor, a psychiatrist, a psychologist, a counselor, a teacher, or another individual providing care/intervention for the child. We will ask for consent to approach these individuals to ask for their participation in the research; the burden on these individuals will be minimal. We will aim to gather information about the impact of the DHI on information-sharing, decision-making, and collaboration at multiple points of care. We will aim to recruit at least two individuals representing additional points of care so that for each child, we have at least three points of care (e.g., home, school, mental health therapist) in addition to direct engagement with the patient (child).

#### Inclusion and exclusion criteria

Our inclusion criteria are as follows: 1) DSM-5-TR diagnosis of ADHD (including Predominantly Inattentive, Predominantly Hyperactive/Impulsive, or Combined types) through prior medical or psychological evaluations at the time of admission to the program, 2) ability to complete questionnaires and use an app in English (Spanish and other languages will be developed at a later time and are not feasible for the initial RCT), 3) reported IQ of at least 80 in order to ensure that the participant has the cognitive skills needed to use the app, and 4) parent/guardian available to consent and provide feedback in English.

Children will be included regardless of treatment status during recruitment and throughout the study (with or without medication and ongoing psychotherapy). Parents will be asked several questions at each assessment related to treatment, including whether the child is taking medications (with medications and dosage recorded, if applicable), whether the child is receiving psychotherapy (and frequency during the study period), and whether the child is receiving any other services (e.g., school-based occupational, speech, or educational therapies). These factors will be recorded in the database to examine any potential differences in outcomes based on existing treatment services. We will not ask parents to change any treatment protocols at enrollment or postpone any outside treatment services once enrolled.

Failure to meet any of the inclusion criteria will result in exclusion. There are no additional exclusion criteria, as we aim to recruit a sample that is generally like the community's population of children with ADHD to enhance the generalizability of the DHI.

#### Assessments

Participants will have four assessments: Baseline (1 week before the start of the intervention period), Week 8 of the intervention period, Week 16 of the intervention period (post-treatment), and 8 weeks later (follow-up). Delayed treatment (DT) participants will have an additional assessment that follows the waitlist period (Pre- Intervention). This design will create a starting point for future studies of longer-term outcomes that are presently beyond the scope of the proposed study. Completing the study measures will require up to one hour for the child and parent at each assessment. For clinicians and educators, completing the questionnaires will require approximately 20 min at each time point. In addition, at each assessment time point, we will conduct semi-structured interviews (~ 30 min each) with participants focused on issues specific to different points of care and experiences with the DHI. Assessments will take place within a 7-day window at each designated timepoint. We will schedule them at a time convenient to participants and will offer remote/virtual platforms to increase adherence.

#### Study sample size

A priori sample size calculation was performed for a parallel group design with four repeated measures. A total sample size of 60 children was identified as sufficient for detecting medium group differences with a type I error rate set to 5% and power set to 81% in response to the intervention across the 4-time points (G*power V3.1.9.6). In our study, we will recruit 60 children (ages 8–12) with ADHD and randomly assign them to one of four groups: immediate intervention, immediate active control, delayed intervention, and delayed active control, with 15 children in each group. For an R33 pilot clinical trial, we aimed for power > 80% as this would allow us to detect trends in outcomes that would inform future research, including a fully-powered randomized clinical trial.

### Quantitative data collection

We will collect quantitative data using (1) well-validated research measures and (2) data gathered from the DHI itself. In this way, we can triangulate the phenomena we observe and create robust models for self-regulation, response to various contextual cues, and our approach's feasibility, acceptance, and potential impact. Standardized outcome measures (see Table 1) will be administered at each assessment timepoint. Quantitative data from outcome measures will be collected to examine both proximal and distal outcomes in a longitudinal repeated measures design. Measures will assess behavioral mechanisms (e.g., self-regulation and self-monitoring), clinical outcomes (e.g., ADHD symptoms), and process outcomes (e.g., patient or provider experience with treatment).

**Table 1:** Standardized quantitative outcome measures

Outcome	Data Sources	Instrument	Description
Child Self- Regulation *(primary outcome)*	Child, Parent, Teacher, Researchers	The Behavior Assessment System for Children (BASC-3) – Self, Parent, and Teacher Reports	BASC-3—a validated and nationally normed self-report scale of self-regulation, emotional, and behavioral symptoms that has been shown to produce reliable scores and has national norms for various symptom subscales
Parent Self- Regulation *(primary outcome)*	Parent	BASC-3 Parenting Relationship Questionnaire (PRQ)	The BASC-3 PRQ includes items that address Parent Self-Regulation, Parenting Confidence, and Parenting Stress
Self-Efficacy *(secondary outcome)*	Child	BASC-3 Self-Report	Self-ratings on self-efficacy, a dimension reflecting competence, capability, and problem-solving. The BASC-3 has three scales that address this domain, which will be examined: Sense of Inadequacy, Self-esteem, and Self-Reliance
Parent–Child Relationship *(secondary outcome)*	Child & Parent	BASC-3 Parenting Relationship Questionnaire (PRQ) BASC-3 Self Report (Relations with Parent scale)	The BASC-3 PRQ is a measure of a parent’s perspective on the parent’s relationship with the child. Our primary focus will be on the Relational Frustration scale The BASC-3 Self-Report measure for children includes scores for a subscale that addresses the parent–child relationship (Relations with Parent). Those scores will be examined to assess the impact of DHI on the parent–child relationship
Engagement in Care* (primary outcome)*	Child & Parent	Patient Activation Measure (PAM) and Parent Patient Activation Measure (Parent PAM)	The 13-item version of the PAM is designed to assess patient knowledge, skill, and efficacy in self-management of their condition. There is also a patient-focused version and a Parent PAM. We will use both in this study to examine the impact on engagement in care for both the child and the parent
Perceptions of Shared-Decision Making and Treatment Collaborations *(primary outcome)*	Child & Parent	Working Alliance Inventory-Short Revised	The Working Alliance Inventory-Short Revised is a 12-item validated scale that evaluates the working relationship between a client and a clinician
	Provider/Educator	Working Alliance Inventory-Short Revised	The Working Alliance Inventory-Short Revised has a provider version of a 12-item validated scale that evaluates the working relationship between a client and a clinician

Additional quantitative data will be gathered from children through smartwatches and parents via the parent smartphone application. This will include usage data and data measuring treatment adherence (e.g., taking medications as prescribed, completing treatment goals and intervention modules) and brief symptom reports (behavioral and emotional). In addition, we will also collect children's sensor data related to physical activity (e.g., bouts of exercise, step counts), sleep, and heart rate.

#### Planned analysis

Multilevel model analyses will be used to adjust for the non-independence (i.e., autocorrelation or clustering) of repeated observations nested within the same individual mixed effects. Such analysis will examine how outcomes within each patient change over time and how individual trajectories of change differ across patients. For each outcome, a mixed-effect model will be estimated with SAS PROC MIXED to test outcome differences between treatment groups at each repeated assessment and differences in the outcome trajectories. Momentary data collected via the scheduled DHI (CoolTaCo) entries will be aggregated to the weekly level, and generalized linear mixed effect models with Poisson distribution for count data will be estimated using SAS PROC GLIMMIX. Tukey adjusted tests will be used for post-hoc comparisons to evaluate significant changes between two time points, treatment groups, or other paired comparisons when a significant interaction was revealed.

Preliminary analyses will be conducted to identify potential individual characteristics (e.g., gender) to be included as covariates. An important strength of multilevel models is the ability to account for unbalanced and/or missing data (which are common problems in ecological momentary data collection) by not placing restrictions on the number of observations obtained for each individual and allows trajectories across time to be estimated from an individual’s available data.

### Qualitative data collection

Interviews are a leading technique for investigating technological settings in human–computer interaction research [31, 32] and help examine complex settings where technical, behavioral, and social factors intersect [33]. Therefore, we will employ interview-based qualitative research methods to develop a rich understanding of the everyday experience of participants, parents, clinicians, and educators engaging with this novel technology. We will conduct semi-structured interviews with system users, including children, their parents or caregivers, and their clinicians and teachers before, during, and after using the technology. The aim of the interviews is to understand the use and adoption of the CoolTaco, comprehend the potential impact of the app in supporting self-regulation, and gather expectations of the app. The interview questions were jointly developed by the research team, based on questions developed and asked by the team in a previous study [22]. Interviews (administered via Zoom when necessary) will be audio-recorded and transcribed verbatim. Study team members conducting interviews and observations will record a) descriptive notes after each observation period and b) analytic memos with descriptive notes and interview transcripts.

#### Planned data analysis

We will use a mix of deductive and inductive qualitative analyses of our interview data. Our deductive analyses will be driven by our hypotheses around the use of DHI in this space as well as our prior work, in which we found comprehensibility of the data, familial interactions around goals and tracking, and comfort with wearing a device as key themes for deeper exploration. Our inductive analysis will occur throughout the project such that data collection, data analysis, and theory development occur continuously, and each activity overlaps and informs the others [34–36].

### Potential problems and strategies

IRB-approved consent forms will describe potential risks and the investigators' responsibility to minimize those risks. Our team includes licensed clinicians who will monitor participants closely, and any participant who experiences psychological or medical difficulty will be referred for appropriate services. All research team members are trained in protocols for reporting suspected child abuse or neglect and will follow California State laws if a situation arises that requires mandated reporting.

We will gather information about the impact of the DHI on information-sharing, decision-making, and working alliances at multiple points of care. When a child and parent enroll, we will ask them to indicate their primary care points (i.e., a pediatrician or family doctor, psychiatrist, or psychologist). We will recruit at least two individuals representing additional points of care to have three points of care for each child (e.g., home, school, mental health therapist). We will ask for consent to approach these individuals and ask for their participation; the burden on them will be minimal.

Other potential risks include phone loss, data loss due to participants forgetting to charge the phone or sensors, and people other than the participants using the phone. To protect against privacy breaches, we will require that participants set up and use the existing user password system on their smartphones for the duration of the study. We will encrypt the data stored on the phones and in transmission to our servers. We will use existing secure cloud services, such as iCloud services, and enable multi-factor authentication.

## Discussion

Individuals with ADHD have complex needs. Despite improvement in outcomes following cognitive behavioral therapies (CBT) and pharmaceutical treatment, long-term maintenance is a challenge that has not been  addressed by traditional medical approaches. Additionally, ineffective information sharing across points of care creates further barriers to progress. Our RCT will create new techniques and add to the latest technological advances, increase communication, and support self-regulation. The CoolTaCo system will contribute and apply to the mental health field while demonstrating the usefulness of DHI for ADHD. Our innovative research will fill a significant gap in translating early treatment investments and gains into long-term, sustainable outcomes.

The study's results may provide knowledge that will be valuable for designing DHI in the future. One benefit that may accrue to the child and family is the return of assessment results and education about electronic data. Children needing medical or psychological treatment based on the level of risk assessed will also receive referrals. Further, participating may give the participants an altruistic experience in helping others by contributing to research knowledge. Participants may also develop self-regulatory skills or improve treatment outcomes and treatment experiences. Because the risks to participants are minor, the potential benefits far outweigh the risks in this project.

In summary, innovative technologies can bridge the gaps leading to many children's poor outcomes. By coupling smartphones with smartwatches, we can offer a sophisticated yet highly available solution to address existing, failing approaches to communication between points of care and long-term maintenance. Our approach will ensure that failures do not squander significant investment in mental health treatment due to lack of reliable information, ineffective communication, delays in the delivery of necessary treatment, and poor self-monitoring/self-regulation. Instead, children and families will get the interventions they need when needed, leading to long-term positive outcomes for individuals and society.

## Data Availability

We understand that to support the goal of advancing research through widespread data sharing among researchers, investigators are expected to share those data, and we propose to do so via the CERES Center at UC Irvine. We will share the final dataset when the final RCT results are published. The data shared will include de-identified participant variables, group assignment variables, and data from measures of clinical symptoms and behavioral data at all timepoints. We will share results, both positive and negative. We will share data when participants have consented to have it shared. We will work closely with the Open Science group as part of the CERES (ceres.uci.edu) network to ensure that open access, open data, and open methods and sources are prioritized in our work.

## Abbreviations

DHI: Digital health interventions
ADHD: Attention Deficit Hyperactivity Disorder
